# AgRP Neuron-Specific Ablation Represses Appetite, Energy Intake, and Somatic Growth in Larval Zebrafish

**DOI:** 10.3390/biomedicines11020499

**Published:** 2023-02-09

**Authors:** Chiu-Ya Lin, Kun-Yun Yeh, Hsin-Hung Lai, Guor Mour Her

**Affiliations:** 1 Department of Bioscience and Biotechnology, National Taiwan Ocean University, Keelung City 202, Taiwan; 2 Institute of Biopharmaceutical Sciences, National Yang Ming Chiao Tung University, Taipei 112, Taiwan; 3 Division of Hemato-Oncology, Department of Internal Medicine, Chang Gung Memorial Hospital, Keelung City 204, Taiwan

**Keywords:** AgRP neuron, neuronal ablation, orexigenic, hypothalamus, appetite

## Abstract

Neuronal circuits regulating appetite are dominated by arcuate nucleus neurons, which include appetite-promoting and -suppressing neurons that release the orexigenic neuropeptide agouti-related protein (AgRP) and anorexigenic neuropeptide pro-opiomelanocortin, respectively, to compete for melanocortin receptors to modulate feeding behavior. In this study, we expressed novel *agrp* promoters, including different lengths of the 5’ flanking regions of the *agrp* gene (4749 bp) in the zebrafish genome. We used the *agrp* promoter to derive the enhanced green fluorescent protein (EGFP)-nitroreductase (NTR) fusion protein, allowing expression of the green fluorescence signal in the AgRP neurons. Then, we treated the transgenic zebrafish AgRP4.7^NTR^ (Tg [*agrp-EGFP-NTR*]) with metronidazole to ablate the AgRP neurons in the larvae stage and observed a decline in their appetite and growth. The expression of most orexigenic and growth hormone/insulin-like growth factor axis genes decreased, whereas that of several anorexigenic genes increased. Our findings demonstrate that AgRP is a critical regulator of neuronal signaling for zebrafish appetite and energy intake control. Thus, AgRP4.7^NTR^ can be used as a drug-screening platform for therapeutic targets to treat human appetite disorders, including obesity. Furthermore, the unique *agrp* promoter we identified can be a powerful tool for research on AgRP neurons, especially AgRP neuron-mediated pathways in the hypothalamus, and appetite.

## 1. Introduction

The arcuate nucleus (ARC) is located in the hypothalamus of the brain. Previous studies have revealed that ARC is crucial for food intake and energy regulation. This includes the appetite-promoting agouti-related protein (AgRP) neurons and the appetite-suppressing pro-opiomelanocortin (POMC) neurons, which can affect neural signal transmission and alter feeding behavior by controlling downstream melanocortin receptors. In the melanocortin system, leptin binds to the leptin receptor, activating POMC neurons, while inhibiting AgRP neurons [1,2,3,4,5]. Leptin and insulin receptors are highly abundant in AgRP and POMC neurons. When these receptors are activated by leptin and insulin, they can trigger the appetite control pathway of cells in the ARC [6,7,8].

AgRP is a neuropeptide produced by AgRP neurons in the ARC of the central nervous system [9,10]. AgRP has been shown to enhance hunger, slow metabolism, and lower energy consumption [10,11]. Under normal conditions, as the number of adipocytes decreases, the production of insulin and leptin is inhibited [12,13], allowing AgRP neurons to be activated and produce AgRP and neuropeptide Y (NPY) to convey body hunger cues that boost appetite, slow the metabolic rate, and limit energy consumption [9,14]. Appetite is repressed by leptin [15] and insulin [16] and triggered by ghrelin [17,18]. AgRP can be used as a competitive antagonist to suppress α-melanocyte-stimulating hormone (α-MSH) and increase appetite by attaching to the melanocortin 4 receptor (MC4R) and preventing α-MSH from binding to MC4R [19,20,21,22,23].

Mice lacking in the *agrp* gene are slimmer and exhibit severe hypophagia [24,25]. Ablation of AgRP neurons via administration of diphtheria toxin directly induces the *Fos* proto-oncogene, AP-1 transcription factor subunit, and gliosis, leading to appetite suppression [26,27]. Similarly, ablation of AgRP1 neurons in larval zebrafish resulted in decreased food intake [28]. The zebrafish melanocortin system also influences somatic growth [29]; morpholino-based *agrp* knockdown leads to lower expression of the growth hormone 1 (*gh1*) gene and shorter body length [23], while transgenic overexpression of AgRP [30] or deletion of α-MSH [31] shows the opposite effects.

Most previous studies on AgRP neurons in zebrafish used the gene knock-in technique to insert the target sequence before the *agrp* transcription start point (such as the AgRP–ires–Cre system) [26,32,33] or a bacterial artificial chromosome library containing the 5’-flanking region of *agrp* [28,34,35,36] to generate transgenic lines. However, the approach described in the literature has several limitations for *agrp*-related research, including low transmission rates, limited flexibility of transgenic constructs, and lack of switchability of transgenic genes. In this study, we sought to identify a novel *agrp* promoter for use in zebrafish AgRP neurons.

This study employed a nitroreductase (NTR) cell ablation system in order to test the functionality of the cloned agrp promoters [26]. NTR can convert the prodrug metronidazole (MTZ) into a cell-autonomous DNA crosslinking agent, thereby initiating apoptosis in the target cell of interest [37,38]. We generated AgRP4.7^NTR^-transgenic zebrafish and obtained NTR/MTZ-ablated AgRP neurons in the hypothalamus of zebrafish. Since AgRP neuron-specific ablation represses appetite and energy intake, resulting in *agrp* and other orexigenic gene depression, AgRP4.7^NTR^ is a novel model for pharmacological studies to investigate these functions and network reorganization as orexin input is lost. In addition, we identified a unique *agrp* promoter that can be used as a potent tool for evaluating AgRP neurons, facilitating the investigation of AgRP neuron-mediated pathways in the hypothalamus, which will be useful for appetite research in the future.

## 2. Materials and Methods

### 2.1. Generation and Maintenance of Transgenic Zebrafish

In this study, the following transgenic zebrafish lines were developed and used: (a) AgRP4.7^NTR^ (Tg [*agrp*-enhanced green fluorescent protein [*EGFP*]-*NTR*]), which showed AgRP neuron-specific expression of EGFP-NTR fusion protein driven by the 4.7 kb *agrp* promoter; (b) POMC^NTR^ [Tg (*pomc-EGFP-NTR*)], which showed POMC neuron-specific expression of EGFP-NTR fusion protein driven by the *pomc* promoter [39]; and (c) β-act^NTR^ [Tg (*β-act-EGFP-NTR*)], which showed global EGFP-NTR fusion protein expression under the control of the *β-actin* promoter. All animal studies were approved by the Institutional Animal Care and Use Committee (IACUC) guidelines (approval ID: 1110209).

Transgenic zebrafish were maintained in a controlled environment with a 14/10-h light–dark cycle at 28 °C. All procedures were conducted in accordance with the IACUC guidelines of the National Yang Ming Chiao Tung University, Taipei, Taiwan.

### 2.2. Promoter Cloning

The 4.7 kb *agrp* promoter used in this study comprised a 5′ flanking region (4749 bp) at the transcription start site of the *agrp* gene and a partial 5′ coding region with an in-frame 9 bp downstream of the *agrp* translational start site, amplified with Q5 High-Fidelity DNA Polymerase (NEB, Ipswich, MA, USA) and the following primers: 5′-end primer, 5′-GTCGACCTCGAGGCTCTGCAAGTGAAGCTTCAGGTC-3′; and 3′-end primer, 5′-CACTAGTCCCAGCATCATAATCACTCAGACTCA-3′. The amplified 4.7 kb *agrp* promoter was purified using a FavorPrep GEL/PCR Purification Mini Kit (FAVORGEN, Ping Tung, Taiwan), according to the manufacturer’s instructions.

For isolation of the 5′ truncated sequences of the 4.7 kb *agrp* promoter, the 2.7, 3.4, and 4 kb *agrp* promoters were generated via polymerase chain reaction (PCR) amplification using the 3′-end primer 5′-CACTAGTCCCAGCATCATAATCACTCAGACTCA-3′, along with primers specific to different regions of the 5′-end *agrp* promoter. The specific primers for amplification of 2.7, 3.4, and 4 kb *agrp* promoters were AgRP2.7 (5′-GTCGACCTCGAGAATGAGGGAAGTTGTGGATACCCG-3′), AgRP3.4 (5′-GTCGACCTCGAGCTCTCACCAGCCCCTCTAGATAAC-3′), and AgRP4.0 (5′-GTCGACCTCGAGGTGCCTTGACCATTTAAATGTGAC-3′), respectively. All PCR products were cloned into the pGEM-T Easy vector (Promega, Madison, WI, USA).

### 2.3. Transcription Factor-Binding Site Prediction

The potential transcription factor-binding site of the cloned zebrafish *agrp* promoter was predicted using Table Browser “https://genome.ucsc.edu/cgi-bin/hgTables” (accessed on 20 October 2022). The datasheet on this website is based on the JASPAR database (version 2022) “https://jaspar.genereg.net/” (accessed on 20 October 2022).

### 2.4. MTZ Treatment

MTZ was administered to the embryos and juvenile zebrafish in a 6-well plate and a 3 L tank, respectively, at a concentration of 10 mM (Sigma-Aldrich, Burlington, MA, USA), with a daily water change. The embryos were treated with MTZ beginning at 2 days post-fertilization (dpf) and ending at 5 dpf. However, for long-term treatment after 7 dpf, the treatment concentration is reduced to 1 mM.

### 2.5. Quantitative Reverse Transcription PCR (RT-qPCR)

Total RNA was extracted from zebrafish tissue using TRIzol reagent (Thermo Fisher Scientific, USA) and column-purified with EasyPure Total RNA Spin Kit Tissue (Bioman, New Taipei City, Taiwan), per the manufacturer’s instructions. Then, 100 ng of purified RNA was reverse-transcribed with a first-strand cDNA synthesis kit (K1691; Thermo Fisher Scientific). For real-time reverse transcriptase quantitative PCR (RT-qPCR), a One-Step Real-Time PCR System (Applied Biosystems, Foster City, CA, USA) was used. Table S1 lists the genes and their corresponding primer sequences used in this study. All investigated gene mRNA expression levels were normalized to *bactin1* levels.

### 2.6. Whole Mount In Situ Hybridization (WISH)

The gene-specific probes used in WISH were cloned by PCR into a pGEM-T Easy vector (Promega, Madison, WI, USA) using the primers listed in Table S2. Antisense probes were created in vitro using a DIG RNA Labelling kit (SP6/T7; Roche Applied Science, Penzberg, Germany). WISH was performed per the procedure described in our previous study [40].

### 2.7. Quantification of Food Intake

To quantify the food intake by zebrafish, the method described by Shimada et al. [41] was used after modifications. Fluorescently tagged paramecia used as the larval fish meal were produced with the lipophilic tracer 4-(4-(didecylamino) styryl)-*N*-methylpyridinium iodide (4-Di-10-ASP; Invitrogen, Carlsbad, CA, USA). To facilitate free swimming, the fish were fed in 8 dpf in a 6-well plate. The larvae were sedated 1 h after feeding. After removing the leftover paramecia by washing twice, groups of five larvae were placed in an anesthetic solution in a 96-well round-bottom black plate. An enzyme-linked immunosorbent assay reader (Bio-Tek Synergy HT in fluorescence area scan mode 9 × 9 multipoint/well, 0.1 s/point) with a fluorescein filter set was used to measure the intra-abdominal fluorescence signal (excitation wavelength, 485 nm; emission wavelength, 590 nm) [31].

### 2.8. Whole-Mount Oil Red O Staining

Zebrafish larvae were fixed overnight at 4 °C in PBS (GeneMark, Taichung, Taiwan) containing 4% paraformaldehyde. Wild-type (WT) and AgRP4.7^NTR^ larvae were distributed in equal numbers into 2 mL tubes and were washed twice (10 min each) with PBS. The larvae were pre-stained with 60% isopropyl alcohol for at least one hour. Thereafter, the larvae were stained with 0.5% Oil Red O solution (Sigma-Aldrich, Burlington, MA, USA) for two hours at 4 °C and then rinsed in PBS. The larvae were observed using a bright-field dissecting microscope (Stemi 305; Carl Zeiss AG, Oberkochen, Germany) while being preserved in 70% glycerol.

### 2.9. Statistical Analysis

Except for the quantitative body length data in the blind test experiment, which were shown as box plots, all the other data are presented as the mean ± standard error of the mean (SEM). For statistical purposes, T-TEST analysis was used. All analyses were conducted using the GraphPad Prism 9.0 program (GraphPad, San Diego, CA, USA). Differences were considered statistically significant at *p* < 0.05.

## 3. Results

### 3.1. Generation of Transgenic AgRP4.7^NTR^ Zebrafish Lines

**Figure 1:**
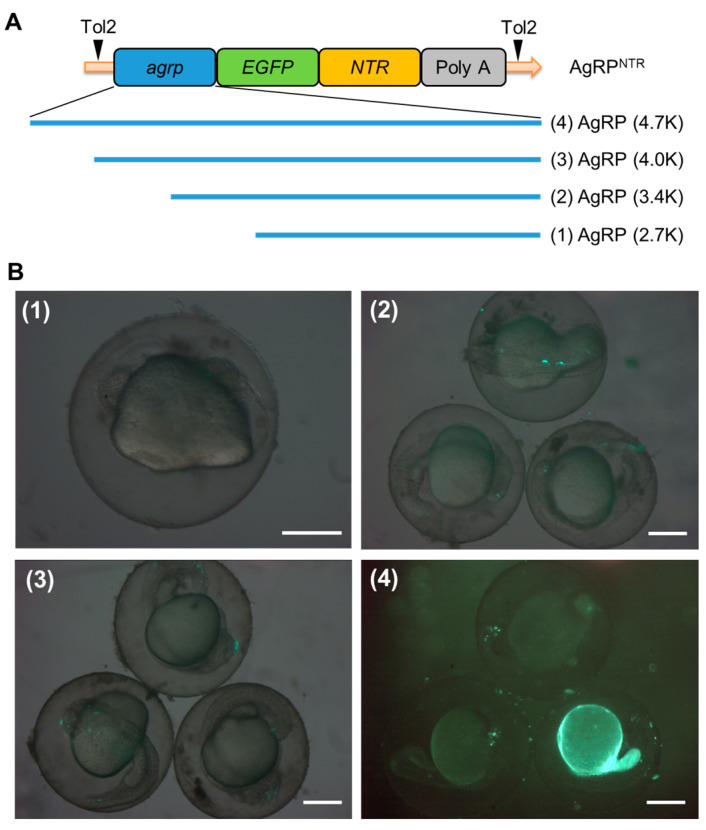
Transient expression analysis of the *agrp*-enhanced, green, fluorescent protein-nitroreductase (*agrp-EGFP-NTR)* deletion constructs. (**A**) Schematic diagram of the DNA construct used to generate AgRP^NTR^ (Tg[*agrp-EGFP-NTR*]) transgenic zebrafish. AgRP (4.7K)-EGFP-NTR contains a 4749 bp 5′ flanking region of the *agrp* gene, and EGFP-NTR fusion has an in-frame 24 bp region downstream of the *agrp* translational start site. (**B**) Each deletion construct was generated as described in Section 2.2 for functional analyses of the promoter. A total of 4.6 nL (15 ng/µL) of the deletion constructs for (**1**) AgRP (2.7K)-EGFP-NTR, (**2**) AgRP (3.4K)-EGFP-NTR, and (**3**) AgRP (4.0K)-EGFP-NTR and the full-length construct for (**4**) AgRP (4.7K)-EGFP-NTR were microinjected into one-cell stage embryos. EGFP signal was observed in 36 h post-fertilization (hpf) embryos injected with these constructs via fluorescence microscopy. Scale bar: 200 µm.

Firstly, we amplified 4 distinct lengths of the 5’ flanking region of the *agrp* gene from the genomic PCR amplicons: AgRP (2.7K), AgRP (3.4K), AgRP (4.0K), and AgRP (4.7K). Then, we created four *agrp-EGFP-NTR* constructs and used them to generate germline-transmissible transgenic zebrafish lines to verify the tissue specificity of the *agrp* promoter and achieve permanent green fluorescence expression in zebrafish AgRP neurons (Figure 1A).

The transgenic fish lines were established following the general Tol2 transposon protocols [42]. The implanted embryos were microscopically evaluated at 36 hpf, assessed for their fluorescence rate (Table 1), photographed (Figure 1B), reared to sexual maturity, and screened for prospective founders. The fluorescence of the five dpf progeny of the founder fish was examined and evaluated using microscopy after they were mated with WT fish.

The transient expression ratio of EGFP in 36 hpf larvae was determined by injecting embryos with constructs containing the 5’ flanking region of different lengths of the AgRP promoter. Embryos injected with the AgRP (4.7K), AgRP (4.0K), and AgRP (3.4K) constructs showed EGFP expression in the anterior region of the brain or trunk of the larvae (Figure 1B). The percentage of fluorescence ratio in AgRP (4.7K) was considerably higher than that of the other groups (Table 1). None of the embryos injected with AgRP (2.7K) showed EGFP expression. This suggested an insufficient length of the promoter region for transcriptional factors to bind in this group. Moreover, the AgRP (4.7K) promoter-driven transgenic construct (AgRP4.7^NTR^) has become the most promising form that could produce functional transgenic fish with stable EGFP-NTR fluorescence in the AgRP neurons of zebrafish.

### 3.2. EGFP-NTR Fusion Protein Causes Target Neural Cell Ablation

We adopted a previously reported NTR cell ablation system in this study [26,43]. We fused *EGFP* with bacterial *NTR* to form the *EGFP-NTR* gene fusion, speculating that its expression would not only produce green fluorescence but also have NTR activity to induce cytotoxicity via MTZ treatment. To confirm EGFP-NTR protein activity in vivo, we used the ubiquitous *β-actin* promoter to drive the EGFP-NTR fusion protein and performed a microinjection test in fertilized zebrafish embryos (Figure S1A). The larvae with this construct showed a green signal in muscle tissues; however, the green fluorescence was quenched by MTZ treatment (Figure S1B).

**Figure 2:**
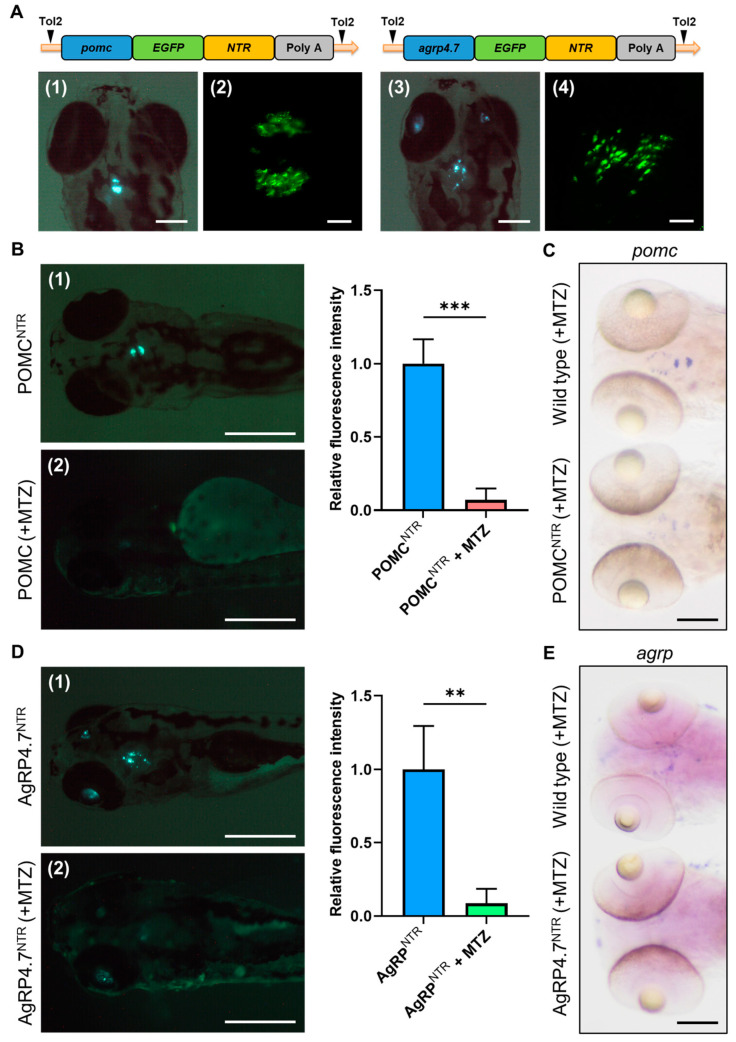
Establishment of POMC^NTR^ and AgRP4.7^NTR^ transgenic fish and neuron ablation tests. (**A**) Schematic diagram of the DNA construct used to generate POMC^NTR^ and AgRP4.7^NTR^ transgenic zebrafish lines. Fluorescence images of (**1**) POMC^NTR^ and (**3**) AgRP4.7^NTR^ larval head at 5 days post-fertilization (dpf) (Scale bar: 200 µm). Confocal images of (**2**) pro-opiomelanocortin (POMC) neurons of POMC^NTR^ and (**4**) AgRP neurons of AgRP4.7^NTR^ at 5 dpf (Scale bar: 20 µm). (**B**) Left: Fluorescence images of (**1**) POMC^NTR^ transgenic zebrafish larvae at 5 dpf and (**2**) metronidazole (MTZ)-treated POMC^NTR^ transgenic zebrafish larvae at 5 dpf, n = 3. Scale bar: 500 µm. Right: quantification diagram of fluorescence intensity. All values are the mean ± standard error of the mean (SEM), n = 30. *** *p* < 0.001. (**C**) Whole mount in situ hybridization (WISH) assays showing the expression signals of *pomc* transcripts in POMC neurons in wild-type (WT) and POMC^NTR^ larvae at 5 dpf, n = 30. Scale bar: 100 µm. (**D**) Left: Fluorescence images of (**1**) AgRP4.7^NTR^ transgenic zebrafish larvae at 5 dpf and (**2**) MTZ-treated AgRP4.7^NTR^ transgenic zebrafish larvae at 5 dpf, n = 3. Scale bar: 500 µm. Right: quantification diagram of fluorescence intensity. All values reported as mean ± SEM, n = 30. ** *p* < 0.01. (**E**) WISH assay results showing the expression signals of *agrp* transcripts in AgRP neurons in WT and AgRP4.7^NTR^ larvae at 5 dpf. Scale bar: 100 µm.

To ensure the capability of the NTR cell ablation system for neuronal cell ablation, the POMC^NTR^ and AgRP4.7^NTR^ transgenic fish experiments were performed (Figure 2A). After three days of MTZ treatment beginning at 2 dpf, the green fluorescence signals of POMC and AgRP neurons in 5 dpf larvae were quenched (Figure 2B,D). WISH analyses revealed significantly decreased expression patterns of *pomc* and *agrp* compared to those in WT fish (Figure 2C,E). As a result, the POMC and AgRP neurons were efficiently ablated by the NTR cell ablation system. Moreover, the AgRP4.7^NTR^ transgenic fish line had a distinct fluorescence pattern that matched the site of AgRP neuron fluorescence [36] or the in situ hybridization pattern [44], as described in the literature.

### 3.3. The 4.7 kb Zebrafish agrp Promoter Has Several Essential Transcription Factor-Binding Sites That Are Exclusively Expressed in the AgRP Neuron

**Figure 3:**
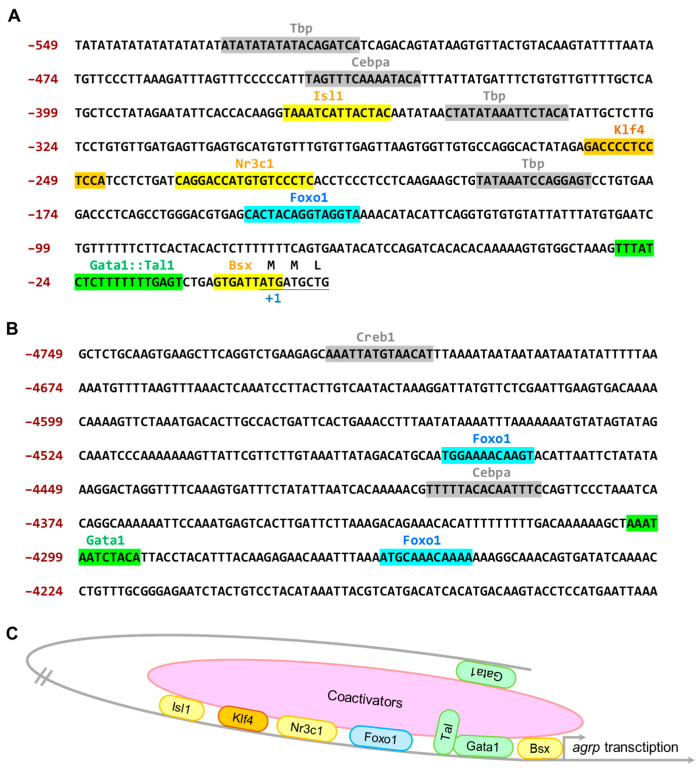
The sequence and potential response elements in the 4.7 kb *agrp* promoter of zebrafish. The nucleotide sequence was downloaded from the Ensembl website “https://asia.ensembl.org/Danio_rerio/Info/Index” (accessed on 2 May 2022). (**A**) The translational start codon (ATG) is underlined and shown as position +1. Nucleotides are numbered on the right, starting with the translational codon. Potential binding motifs in the proximal region are also marked. (**B**) Sequences of the putative *agrp* regulatory element. The predicted binding motif within a distal region of *agrp* promoter. Potential binding motifs are marked. (**C**) Hypothetical model of *agrp* transcription activation in zebrafish. The proteins shown in the figure are: TATA-box-binding protein (Tbp), CCAAT/enhancer-binding protein alpha (Cebpα), ISL LIM homeobox 1 (Isl1), Kruppel-like factor 4 (Klf4), nuclear receptor subfamily 3 group C member 1 (Nr3c1), forkhead box O1 (Foxo1), GATA-binding protein 1 (Gata1), T cell acute lymphocytic leukemia 1 (Tal1), and brain-specific homeobox (Bsx).

Since no information was available on the transcription factor datasheet for the zebrafish *agrp* promoter in the literature, we used the Table Browser website “https://genome.ucsc.edu/cgi-bin/hgTables” (accessed on 20 October 2022) to search the 5′ flanking region of the transcription start site (4749 bp in length) of the *agrp* gene in the zebrafish chromosome position: chr7: 35,083,136–35,087,884. As a result, many potential transcription factor-binding sites were identified in the 4.7 kb *agrp* upstream region. In the proximal region (Figure 3A), several predicted sites for developmental regulatory factors were found, including consensus motifs of brain-specific homeobox (Bsx) [18,45,46,47], GATA-binding protein 1 (Gata1) [48], forkhead box O1 (Foxo1) [18,49], nuclear receptor subfamily 3 group C member 1 (Nr3c1) [45,46], Kruppel-like factor 4 (Klf4) [50,51,52], and ISL LIM homeobox 1 (Isl1) [45,53], all of which are considered key AgRP transcription factors or enhancers in mice and humans.

As a result, we surmised that the 4.0–4.7 kb promoter region is an important regulatory area of the *agrp* promoter (Figure 3B), regulating the essential promoter activity of *agrp*, including the consensus motifs of cAMP-responsive element-binding protein 1 [18,51], Gata1, Foxo1, and Isl1. The findings of this study suggest that this region may play an essential role in enhancing *agrp* gene expression (Figure 3C).

### 3.4. Targeted Ablation of AgRP Neuron Represses Food Intake and Body Growth in Zebrafish Larvae

**Figure 4:**
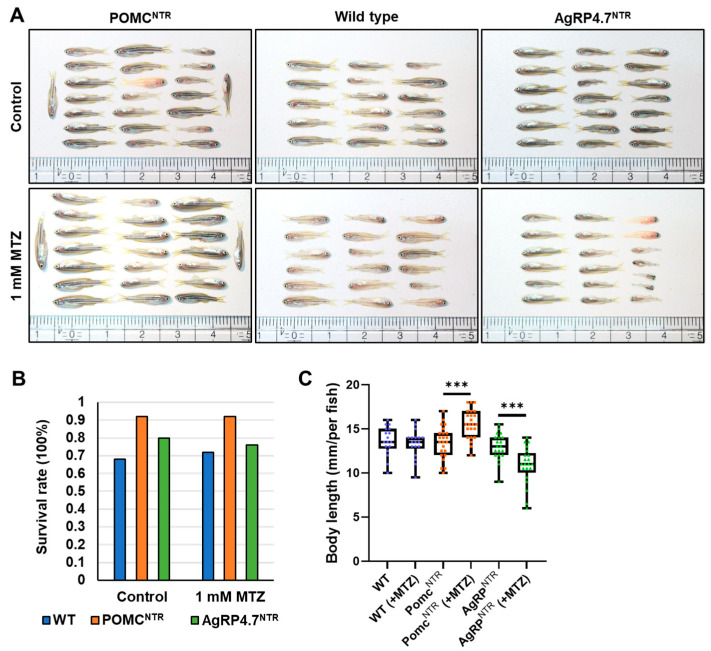
Blind test experiment of targeted neuron ablation transgenic fish. (**A**) Lateral views of POMC^NTR^, WT, and AgRP4.7^NTR^ six-week-old larvae. (**B**) Survival rate of each group. (**C**) Statistical analysis of body length (jaw to caudal fin) in six-week-old POMC^NTR^, WT, and AgRP4.7^NTR^ larvae. Data are shown as box plots including median (line), interquartile range (box), and 95% confidence interval (whiskers). *** *p* < 0.001.

A blind test experiment was performed to validate whether the POMC^NTR^ and AgRP4.7^NTR^ transgenic fish can deplete POMC or AgRP neurons under MTZ treatment to accomplish neuronal ablation and further impact appetite control. In the experiment, 2 fish groups were set up, each with 25 POMC^NTR^, AgRP4.7^NTR^, and WT fish for mixed feeding. During the experiment, one control group received no medication. In contrast, the other experimental group received 1 mM MTZ beginning at 1 week of age, and the drug was constantly administered for 4 weeks until the fifth week, followed by a halt for 1 week until the sixth week (Figure 4A).

The two groups of experimental fish were first observed using a fluorescence microscope to differentiate POMC^NTR^, AgRP4.7^NTR^, and WT. For each group, the survival rate (Figure 4B) and body length were measured. The values of both parameters were calculated and plotted as a statistical graph (Figure 4C). In the experimental group treated with MTZ, POMC^NTR^ fish had a larger body size, while AgRP4.7^NTR^ fish were relatively thin and small.

**Figure 5:**
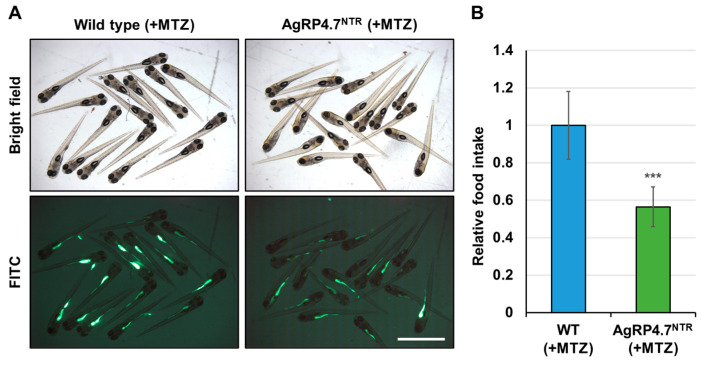
Qualitative food intake assay for MTZ-treated WT and AgRP4.7^NTR^ larvae. (**A**) Side views of 8 dpf larvae were observed under fluorescence lighting. MTZ-treated WT and AgRP4.7^NTR^ larvae were cultivated with 4-Di-10-ASP-labeled paramecia for 1 h before observing the fluorescent contents in their guts. Scale bar: 2 mm. (**B**) Relationship between the relative amount of paramecia and fluorescence intensities of ingested paramecia in zebrafish. All values are represented as the mean ± SEM, n = 50. *** *p* < 0.001.

Next, we employed a previously established nursing regimen to generate fluorescent food, consisting of larval fish meal with lipophilic tracer 4-Di-10-ASP-labeled paramecia [31], to evaluate the pattern of food consumption at the larval stage of the MTZ-treated AgRP4.7^NTR^. The larvae consumed luminous food, which caused strong fluorescence signals to build up in their gut (Figure 5A).

**Figure 6:**
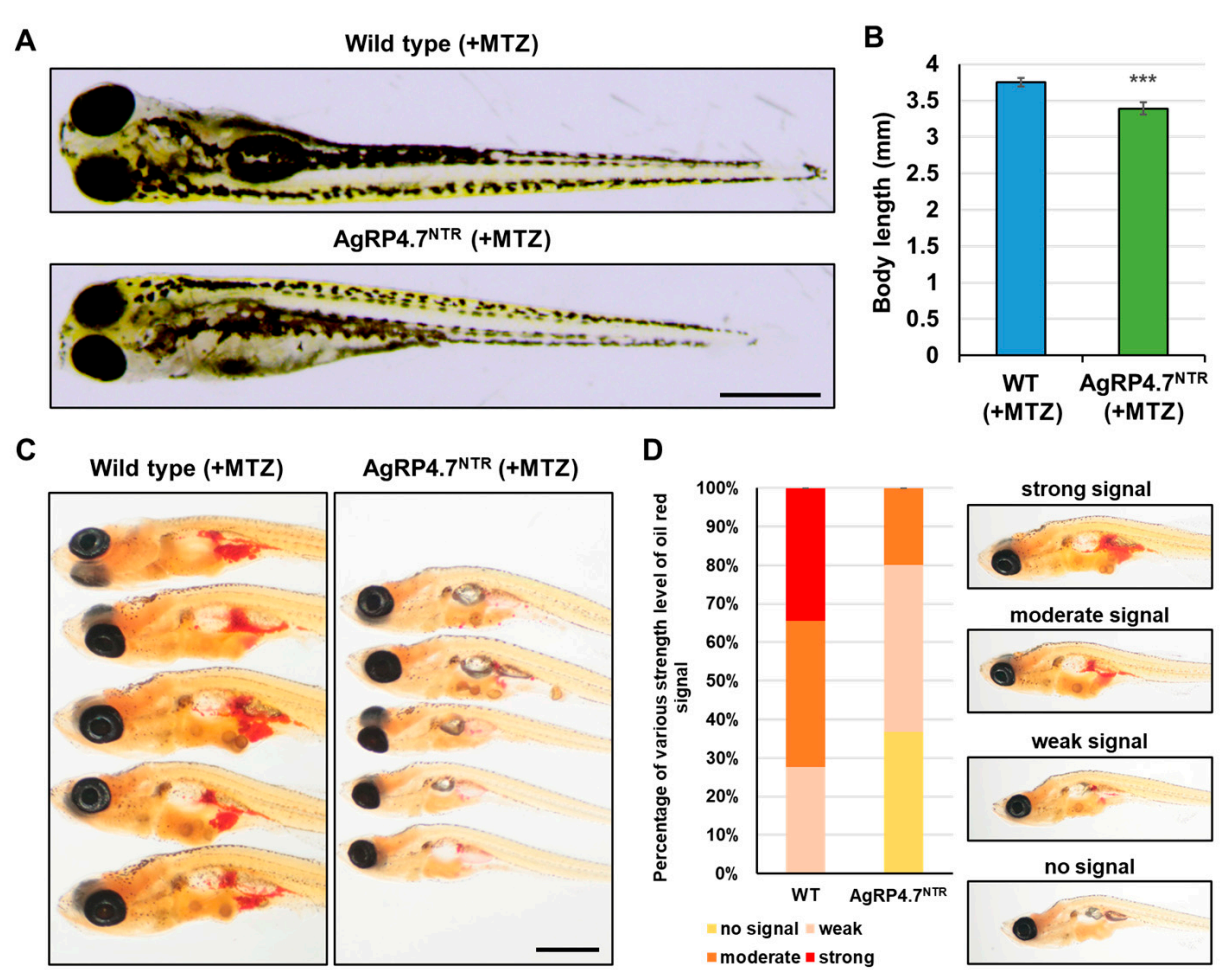
AgRP neuron ablation results in the inhibition of somatic growth and adipocyte formation. (**A**) Lateral view of MTZ-treated WT and AgRP4.7^NTR^ larvae at 8 dpf. Scale bar: 500 µm. (**B**) Statistics of body length measurement between MTZ-treated WT and AgRP4.7^NTR^ larvae at 8 dpf. All values are reported as mean ± SEM, n = 30. *** *p* < 0.001. (**C**) Lipid droplet accumulation was observed via whole-body Oil Red O staining of MTZ-treated WT and AgRP4.7^NTR^ larvae at 21 dpf. Scale bar: 1 mm. (**D**) Left: Distribution of lipid droplets in MTZ-treated WT and AgRP4.7^NTR^ larvae at 21 dpf, n = 30. Right: Intensity grading diagram.

At 8 dpf, the AgRP4.7^NTR^ larvae treated with MTZ showed anorexia, with a 0.44-fold decrease in food consumption compared with WT zebrafish (Figure 5B). Next, we investigated whether growth and energy budget were related to food intake in AgRP4.7^NTR^ and discovered that contemporaneous observations were required to estimate these parameters. Moreover, AgRP4.7^NTR^ transgenic fish showed a considerable decrease in body length (Figure 6A,B). Furthermore, the accumulation of lipid droplets in MTZ-treated AgRP4.7^NTR^ was substantially lower than that in WT larvae (Figure 6C,D).

### 3.5. Defective AgRP Neuron Suppresses the Expression of Orexigenic Genes and Growth Hormones While Increasing the Anorexigenic Gene Expression in Zebrafish Larvae

**Figure 7:**
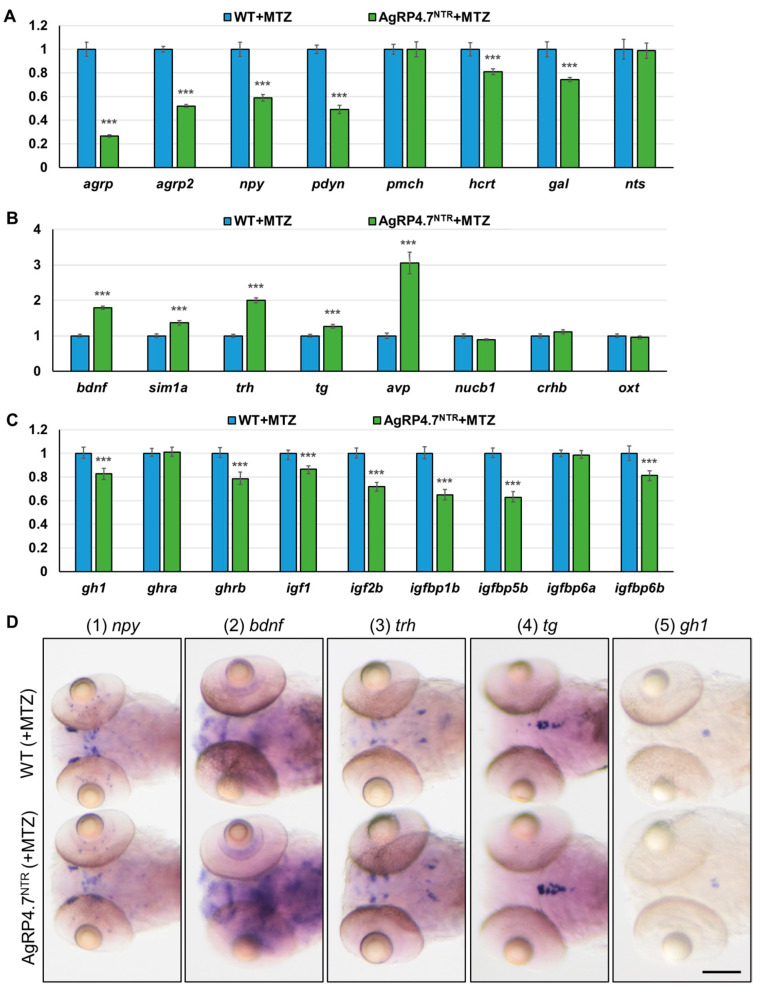
Effects of AgRP ablation on appetite and growth regulation in the brain of zebrafish. Real-time RT-qPCR analysis was used to measure the mRNA expression levels of (**A**) orexigenic genes (*agrp*, *agrp2*, *npy*, *pdyn*, pro-melanin concentrating hormone (*pmch*), *hcrt*, *gal*, and neurotensin (*nts*)); (**B**) anorexigenic genes (*bdnf*, *sim1a*, *trh*, *tg*, *avp*, nucleobindin 1 (*nucb1*), corticotropin releasing hormone b (*crhb*), and oxytocin (*oxt*)); and (**C**) GH/IGF axis genes (*gh1*, *ghra*, *ghrb*, *igf1*, *igf2b*, *igfbp1b*, *igfbp5b*, *igfbp6a*, and *igfbp6b*) in MTZ-treated WT and AgRP4.7^NTR^ larvae at 8 dpf, n = 30. Values are represented as the mean ± S.E.M. *** *p* < 0.001. (**D**) WISH experiment revealed the expression levels and patterns of *npy*, *bdnf*, *trh*, *tg*, and *gh1* in the brains of MTZ-treated WT and AgRP4.7^NTR^ larvae at 5 dpf, n = 30. Scale bars = 100 µm.

Appetite-related gene expression levels in the brains after AgRP neuron ablation were determined using real-time RT-qPCR to analyze the brain gene profiles of MTZ-treated AgRP4.7^NTR^ larvae. To compare the gene expression ratio of appetite-related genes between MTZ-treated WT and AgRP4.7^NTR^ larvae, we examined the expression levels of several critical factors, including orexigenic genes, *agrp*, *agrp2*, *npy* [54,55], prodynorphin (*pdyn*) [56,57], hypothalamic hypocretin/orexin (*hcrt*) [58], and galanin/GMAP prepropeptide (*gal*) [59], which were repressed by AgRP neuron ablation (Figure 7A). In contrast, the levels of anorexigenic genes, including brain-derived neurotrophic factor (*bdnf*) [60,61], single-minded homolog 1-a (*sim1a*) [57,62], thyrotropin-releasing hormone (*trh*) [57,63], thyroglobulin (*tg*) [63], and arginine vasopressin (*avp*) [64,65], were promoted by AgRP neuron ablation (Figure 7B). Additionally, the expression levels of growth hormone (GH)/insulin-like growth factor (IGF) axis genes, including *gh1* [23,29,31], growth hormone receptor a (*ghra*), *ghrb*, *igf1*, *igf2b*, insulin-like growth factor-binding protein 1b (*igfbp1b*), *igfbp5b*, *igfbp6a*, and *igfbp6b,* were decreased by AgRP neuron ablation (Figure 7C).

**Figure 8:**
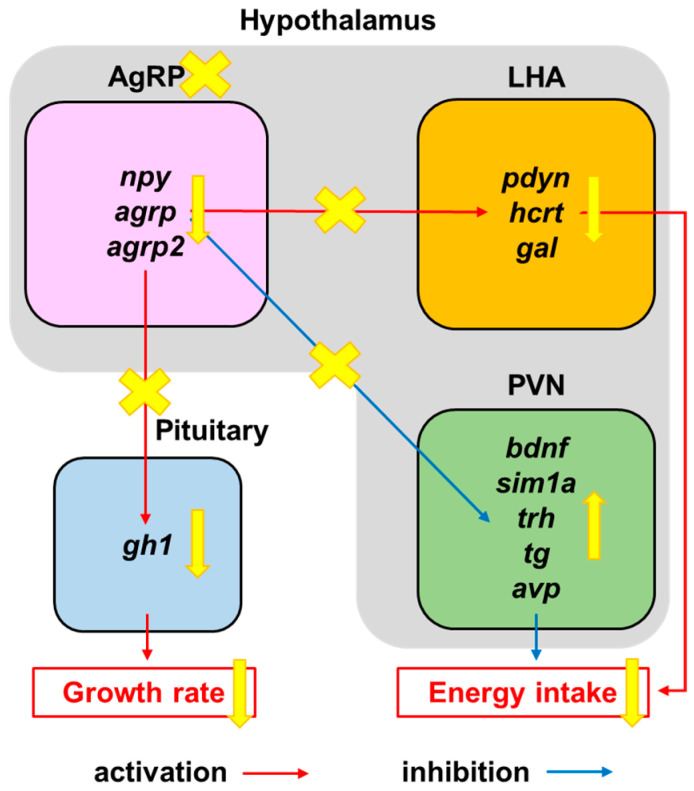
Appetite-regulation pathway under AgRP neuron ablation. Schematic diagram of suppression of orexigenic factors (*pdyn*, *hcrt*, and *gal*) within the lateral hypothalamus area (LHA) and promotion of anorexigenic factors (*bdnf*, *sim1a*, *trh*, *tg*, and *avp*) within the paraventricular nucleus (PVN) via AgRP neuron ablation, leading to appetite and growth suppression.

WISH was used to identify the appetite-related gene expression patterns and visible expression of genes, including *npy*, *bdnf*, *trh*, *tg,* and *gh1*, and the results were consistent with those of real-time RT-qPCR (Figure 7D). Our data showed that most orexigenic and growth hormone mRNA levels were significantly lower in MTZ-treated AgRP4.7^NTR^ than in WT at 8 dpf, and most anorexigenic mRNA levels were markedly higher in MTZ-treated AgRP4.7^NTR^ than in WT at 8 dpf. These results showed that our newly established MTZ-treated AgRP4.7^NTR^ larvae could effectively ablate AgRP neurons, reduce the AgRP neuron peptide signal, and further affect the regulation of downstream brain appetite-related genes, resulting in appetite and growth suppression (Figure 8).

## 4. Discussion

To the best of our knowledge, no stable transgenic lines harboring *agrp* gene promoters have been described previously, and none of the AgRP-specific promoters in zebrafish have been subjected to an in-depth analysis so far. The development of transgenic zebrafish expressing a reporter gene in specific tissues or organs will aid in the study of vertebrate organogenesis, as zebrafish have gained popularity as model organisms. *agrp* gene expression in AgRP neurons in zebrafish has previously been demonstrated [44]. The zebrafish *agrp* promoter region was identified and described in this study.

In this study, we successfully cloned ARC-specific promoters containing either *pomc* [39] or *agrp* and generated transgenic zebrafish with ARC-specific promoter-driven transgenes. Following MTZ treatment of transgenic larvae, the target cells began to undergo apoptosis. For different promoters, such as *β-actin*, *pomc*, and *agrp*, fluorescence signal intensity in the target cell decreased and eventually disappeared. This finding demonstrated that the EGFP-NTR fusion protein can indicate the target area with visible fluorescence and retain cell toxicity. Therefore, it can potentially be used in a cell ablation technique in the future.

Transcription factor-binding site analysis of the 5’ flanking region of the transcription start site of the *agrp* gene showed that several predicted transcription factors and enhancers were involved in the promoter region (–591 to –1 bp and –4749 to –4149 bp). Previous literature suggests that among the transcription factors we identified, ISL1, BSX, and glucocorticoid receptor (GR, known as Nr3c1) bind to the homeodomain response elements (HDREs) and glucocorticoid response element (GRE) to form a ternary complex, which then synergistically stimulates glucocorticoid-directed transactivation of the *agrp* promoter, likely by facilitating the recruitment of transcriptional coactivators to the *agrp* promoter [45]. This hypothesis is also consistent with the findings of the present study.

We also discovered that appetite inhibition in the MTZ-treated AgRP4.7^NTR^ model was associated with decreased hunger signals and inhibited somatic growth, as indicated by the gene profile results in this study. Moreover, we have summarized the importance of melanocortin circuits in controlling hunger. Our results contribute to a better understanding of the primary processes relating AgRP signaling and energy intake in zebrafish.

As a result, the 4.7 kb *agrp* promoter identified in this research will be a powerful tool for in vivo studies. It will make it easy for researchers to set up zebrafish models of human metabolic syndromes, such as appetite disorders and obesity, to find novel target genes involved in appetite regulation and to test medications for treating these disorders.

In addition to mimicking human Prader–Willi syndrome or bdelygmia by affecting the regulatory centers of feeding behavior, we can assess metabolism-related genes, such as peroxisome proliferator-activated receptor gamma [66], diacylglycerol O-acyltransferase 2 [67], and CCAAT enhancer-binding protein alpha [68], and simulate and investigate obesity induced by various factors by overexpressing or knocking out genes in AgRP neurons to identify effective strategies to maintain health, through methods such as drug screening and gene therapy.

## Figures and Tables

**Table 1:** Expression of EGFP in 36 hpf embryos injected with constructs containing the *agrp* promoter sequences.

Construct	No. of Embryo Observed	No. of Embryo with EGFP Expression			Fluorescence Ratio (%)
		Head	Trunk	Total	
AgRP (2.7K)	205	0	0	0	0.00
AgRP (3.4K)	215	1	4	5	2.33
AgRP (4.0K)	221	5	6	11	4.98
AgRP (4.7K)	232	32	11	43	18.53

## Data Availability

The data presented in this study are available upon request from the corresponding author.

## Supplementary Materials

The following supporting information can be downloaded at: https://www.mdpi.com/article/10.3390/biomedicines11020499/s1, Figure S1: Functional test of EGFP-NTR fusion protein, Table S1: Primer sequences used for real-time quantitative polymerase chain reaction (qPCR), Table S2: Primer sequences used for in situ hybridization probes.
