# Supplementary material for: AgRP Neuron-Specific Ablation Represses Appetite, Energy Intake, and Somatic Growth in Larval Zebrafish
Source: Biomedicines. 2023 Feb 9;11(2):499. doi: 10.3390/biomedicines11020499 (PMC9953713; doi:10.3390/biomedicines11020499)
Supplement: Supplementary file 1 [file biomedicines-11-00499-s001.zip › Figure S1. Functional test of EGFP-NTR fusion protein.pdf]

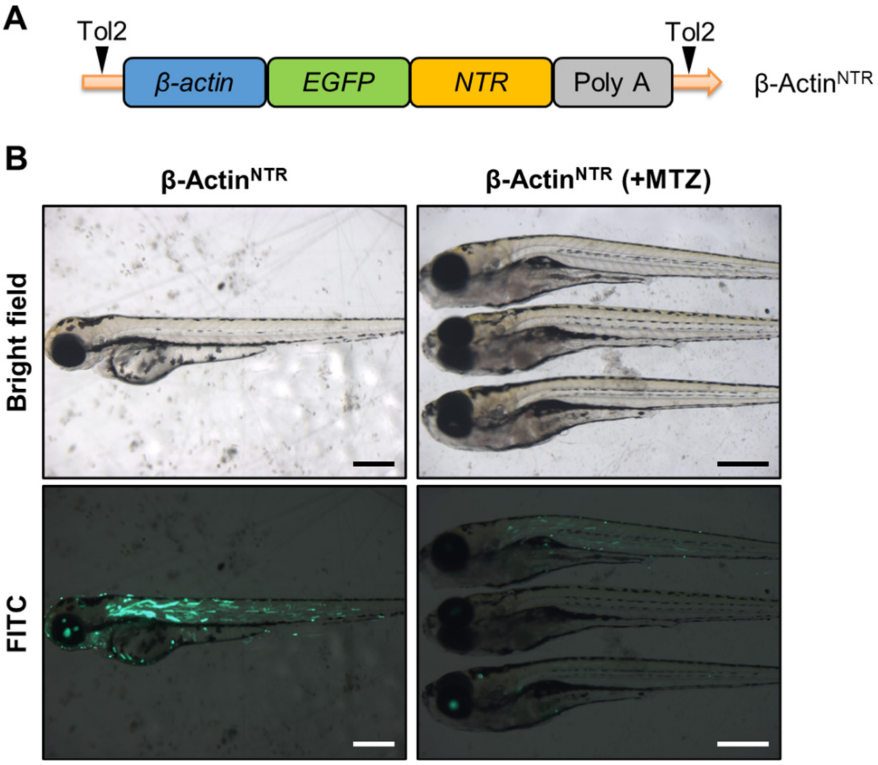

Figure S1. Functional test of EGFP-NTR fusion protein. (**A**) Schematic diagram of the DNA construct used to generate β-Actin^{NTR} (Tg[*β-actin-EGFP-NTR*]) transgenic zebrafish. *β-actin-EGFP-NTR* contains a ubiquitous *β-actin* promoter, and EGFP-NTR fusion has an in-frame 24 bp region downstream of the β-Actin translational start site. (**B**) Fluorescence images of (1) β-Actin^{NTR} transgenic zebrafish larvae at 3 days post-fertilization (dpf) and (2) metronidazole (MTZ)-treated β-Actin^{NTR} transgenic zebrafish larvae at 8 dpf (5 d post-treatment [dpt]). Scale bar: 500 μm.
