# Supplementary material for: AgRP Neuron-Specific Ablation Represses Appetite, Energy Intake, and Somatic Growth in Larval Zebrafish
Source: Biomedicines. 2023 Feb 9;11(2):499. doi: 10.3390/biomedicines11020499 (PMC9953713; doi:10.3390/biomedicines11020499)
Supplement: Supplementary file 1 [file biomedicines-11-00499-s001.zip › Table S1. Primer sequences used for real-time quantitative polymerase chain reaction (qPCR).pdf]

Table S1. Primer sequences used for real-time quantitative polymerase chain reaction (qPCR)

Gene	Accession	Forward Primer	Reverse Primer
bactin1	NM_131031.1	CGAGCAGGAGATGGGAACC	CAACGGAAACGCTCATTGC
agrp	NM_001328012.1	ACACAGTAATCTTCGGCTGG	TCCATTTCAGGCAGTGAGTC
agrp2	NM_001271291.1	AGTTCAGTCCATCACATGCG	TTTCTCCTGATTCCACACTCC
npy	NM_131074.2	AGCACTAAGACACTACATCAACC	GATGAGATCACCATGCCAAATG
pdyn	NM_001001831.1	TGGAGTGGTATGTGTTGGTG	CGGCTCCTTCACACTCTAAAG
pmch	NM_001202542.1	TTCATACTTGCGGACACAGG	TCCATCGTGCTGAATCCATC
hcrt	NM_001077392	TCATGGACTGCACAGCTAAG	CATCTCGTAGAGTTTGCAGGAG
gal	NM_001346239.1	CATTGATAGTCACAGGAGCCTC	CGACATCCTCATCTGCTATCC
nts	XM_021473261.1	GCAATAGAGGATGAGGTTCTGAG	CTTCCAGCCACGAGTCAC
bdnf	NM_001308648.1	AGCATCTGTTGGAGTGTGTG	CAGCTCTCATGCAACTGAAG
sim1a	NM_178222.3	CTGTACATTTCAGAAACGGCG	AAGTGTGAATGATAGGGCTGG
trh	NM_001012365.2	GCGCTCCATCCTCACAC	GCTGTCGCTTCTCCATCC
tg	NM_001329865.1	ACACCTTCAGGATACAACGTG	GCAGCGTTTTCAGGTAAGTG
avp	NM_178293.2	CTACATCCAGAACTGCCCAC	GGCCACACGACATACACTG
nucb1	NM_001045463.1	AAGACCTGGAACTGCTTATCG	GGTCCAAACTCTTCAGGTACTC
crhb	NM_001007379.1	CCAAGGATTTACCAATTACGCAC	CGATGGCTCTACATTCATACGG
oxt	NM_178291.2	CCCATTCGACAGTGTATGCC	ACACGGAGAAGGGAGAAAATC
gh1	NM_001020492	AGAAGGACATGCACAAGGTG	GCAGAAAAGGATAAAGCAGGC
ghra	NM_001083578.1	GGCCGAAAATTCCTTACTGTT	GCTGGCGTTGCTGATTGT
ghrb	NM_001111081.1	GCTGCGCTCTGTTGATAATGT	GGCGGAGGGAGGTGGAT
igf1	NM_131825.2	GCATAGCCACTCTTCCTGTAAG	TCCAACGGTTTCTCTTGTCTC
igf2b	NM_001001815	AACCTGCCAAGTCAGAGAGGG	GGACCTCCTGTTTTAATGCGG
igfbp1b	NM_001098257.2	GTCACAACTCAACGCCATCC	GAACTTCTCTCCCAACGCCT
igfbp5b	NM_001126463.1	CAGGGGAGTGTGTACGAACG	AGTCGTGTCTGGCTTCACTG
igfbp6a	NM_001161401.1	TTCAGGAGGAAGCAGTGTCG	CATCGCTTCTCCTACGGGAC
igfbp6b	NM_001161402.2	CATCGCTTCTCCTACGGGAC	GGGGTCTCGAACTCACCATT
