# Supplementary material for: AgRP Neuron-Specific Ablation Represses Appetite, Energy Intake, and Somatic Growth in Larval Zebrafish
Source: Biomedicines. 2023 Feb 9;11(2):499. doi: 10.3390/biomedicines11020499 (PMC9953713; doi:10.3390/biomedicines11020499)
Supplement: Supplementary file 1 [file biomedicines-11-00499-s001.zip › Table S2. Primer sequences used for in situ hybridization probes.pdf]

**Table S2. Primer sequences used for in situ hybridization probes**

| Gene | Accession | Forward Primer | Reverse Primer |
|---|---|---|---|
| *agrp* | NM_001328012.1 | CTGGGACGTGAGCACTACAGTCTG | CAGCCAATGGTGCACTCTATGCTT |
| *pomca* | NM_181438 | ACAGAGGAGAACATCTTGGAATGC | GCCCACCTTCGTTTCTATGCATGA |
| *npy* | NM_131074.2 | TATCCAACAAAACCCGACAAC | AATGGACACAACACGCATACA |
| *bdnf* | NM_001308648.1 | GTCATTGAGGAGTTGCTTGAG | TATCTGCCCCTCTTAATGGTC |
| *tg* | NM_001329865.1 | GGATGGGTTCAGAAGAACATT | GAGGTCTCTGGTAGCGTTTTC |
| *trh* | NM_001012365.2 | ACACAGATGGAGGAGCAGAAC | CTTACCCCAGTGTACAGCAGA |
| *gh1* | NM_001020492 | ATCCGTGTGCAACACCTTCACCAG | CCATGCCTGCTTGATATTTCCTGC |
